# Effects of Cardiotoxins on Cardiac Stem and Progenitor Cell Populations

**DOI:** 10.3389/fcvm.2021.624028

**Published:** 2021-04-27

**Authors:** Andrew J. Smith

**Affiliations:** ^1^Faculty of Biological Sciences, School of Biomedical Sciences, University of Leeds, Leeds, United Kingdom; ^2^Faculty of Life Sciences and Medicine, Centre for Human and Applied Physiological Sciences, Centre for Stem Cell and Regenerative Medicine, School of Basic and Medical Biosciences, Guy's Campus, King's College London, London, United Kingdom

**Keywords:** cardiac stem/progenitor cells, cardiotoxicity, heart failure, regeneration, microvasculature

## Abstract

As research and understanding of the cardiotoxic side-effects of anticancer therapy expands further and the affected patient population grows, notably the long-term survivors of childhood cancers, it is important to consider the full range of myocardial cell types affected. While the direct impacts of these toxins on cardiac myocytes constitute the most immediate damage, over the longer term, the myocardial ability to repair, or adapt to this damage becomes an ever greater component of the disease phenotype. One aspect is the potential for endogenous myocardial repair and renewal and how this may be limited by cardiotoxins depleting the cells that contribute to these processes. Clear evidence exists of new cardiomyocyte formation in adult human myocardium, along with the identification in the myocardium of endogenous stem/progenitor cell populations with pro-regenerative properties. Any effects of cardiotoxins on either of these processes will worsen long-term prognosis. While the role of cardiac stem/progenitor cells in cardiomyocyte renewal appears at best limited (although with stronger evidence of this process in response to diffuse cardiomyocyte loss), there are strong indications of a pro-regenerative function through the support of injured cell survival. A number of recent studies have identified detrimental impacts of anticancer therapies on cardiac stem/progenitor cells, with negative effects seen from both long-established chemotherapy agents such as, doxorubicin and from newer, less overtly cardiotoxic agents such as tyrosine kinase inhibitors. Damaging impacts are seen both directly, on cell numbers and viability, but also on these cells' ability to maintain the myocardium through generation of pro-survival secretome and differentiated cells. We here present a review of the identified impacts of cardiotoxins on cardiac stem and progenitor cells, considered in the context of the likely role played by these cells in the maintenance of myocardial tissue homeostasis.

## Introduction

Managing anticancer therapy side-effects has been an important component of oncotherapy care since it was first developed, with cardiotoxicity as one of the principal side-effects. As anticancer therapy regimens become more effective and patient long-term survival increases, avoidance of cardiotoxicity, and its long-term management have accordingly become of ever-greater importance. Two developments in healthcare continue to drive this: one positive, the other negative. The first is improving cancer patient survival: a review of 40 years of cancer care in England and Wales found that patients had 50% survival 1 year after diagnosis in 1971, which rose to 50% survival (predicted) at 10 years after diagnosis in 2011 (1). The second is that cardiovascular disease remains highly prevalent in the wider population, disease to which cancer survivors are no less susceptible. In fact, as the pathophysiology of cardiotoxicity due to many anticancer drugs involves myocardial ischaemia or coronary artery damage (2), along with recognition that cardiotoxicity may manifest in cancer survivors only in the long term (3), the risk is likely notably higher.

Doxorubicin (DOX) and other anthracyclines are long established in anticancer therapy, used for several cancers of childhood (4), but are also well-recognised as cardiotoxic, and DOX has more recently been identified as a cause of cardiotoxic damage manifesting in the long term [for review, see Ref. (3)]. More recent anticancer therapy classes such as tyrosine kinase inhibitors (TKIs) are more precisely targeted, acting to impair tumour cell proliferation (5), migration (6), and tumour angiogenesis (7) *via* focused actions on tyrosine kinases (acting on a few kinases to a broad range, depending on the individual TKI). However, emerging data indicate cardiotoxic side-effects for these drugs, underlining the importance of considering these in patient management, with comparable cardiotoxicity data emerging for the epidermal growth factor receptor-2 inhibitor trastuzumab (8).

Microvascular injury is an early sign of several cardiac disease processes, and the study of its involvement in cardiotoxicity has grown to generate more complete understanding of the underlying pathophysiology, beyond only mechanisms of injury to cardiomyocytes. Application of DOX to human cardiac microvascular vessel specimens *ex vivo* causes significant reduction in flow-mediated dilation responses in both adult and paediatric vessels, although less extensively in paediatric samples, in addition to loss of acetylcholine-induced vessel constriction responses (9). Microvascular damage is also caused by TKIs, with microvascular dysfunction induced by one TKI with clinically identified cardiotoxicity (10).

Although optimising patient care outcomes is the primary concern, healthcare economic considerations are only realistic, particularly as healthcare demands are ever growing and thus economic resources available are ever more hard pressed to meet them. For all of these reasons, optimising understanding of the pathophysiology of cardiotoxicity and identifying means to avert or minimise its impact is of ever-greater importance.

## Cardiac Stem Cells and Their Role in Tissue Homeostasis

From their discovery in 2003 (11), cardiac stem cells (CSCs), also known as cardiac progenitor cells (CPCs), attracted significant research investment and extensive discussion. The natural first course of investigation was assessing CSCs as a potential source of cardiomyocytes, with multiple papers focused on determining this potential (12–15). Although this knowledge expansion was commendably rapid, the advancement to clinical trials of CSC regenerative potential (16, 17) suffered from this rapidity. Major questions arose over the assumptions about CSCs' mode of regeneration in already-started trials (with retraction of underlying laboratory data undermining one trial). These issues caused cessation of some trials and re-appraisal of likely repair mechanisms in others (18, 19).

The fundamental problem was substantially varied rates of cardiomyogenesis across studies, particularly in the myocardial infarction (MI) setting (20), bringing into question the practical utility of CSCs as a source of new myocardium. As this uncertainty developed, more studies focused on CSCs as generators of protective paracrine effects (21, 22), to be exploited as a cardiac disease therapy (23, 24). This mechanism was later held to be a likely cause of many of the benefits seen in clinical trials (19, 22). Notably, however, in the specific context of diffuse injury (as opposed to post-MI), evidence of cardiomyocyte repair and regeneration was more robust (15, 25), although still contested. One notable diffuse cardiac injury model was DOX treatment *in vivo*: this significantly upregulated cardiomyogenic lineage markers in CSCs and increased new cardiomyocyte formation *in situ*, derived from cells bearing the CSC marker c-kit (26).

One factor contributing to variant findings was dissimilarity in genetic lineage tracing approaches. A study directly comparing transgenic and knock-in-based c-kit-tracing models found significantly reduced c-kit expression in knock-in models (thus a limitation of much evidence contesting CSC cardiomyogenesis) but also that c-kit expression was found in already-formed cardiomyocytes and increased post-injury (thus, a limitation to using c-kit to trace *de novo* cardiomyocytes) (27). For an overview of c-kit genetic tracking to quantify CSC cardiomyogenesis, see Ref. (28). Other studies avoided complications by using a dual-recombination model of c-kit tracing (avoiding Cre-related limitations) (29) or a series of CRISPR/Cas9-based tracking models (30): these studies saw respectively little and no identifiable cardiomyocyte formation from non-myocytes post-MI.

Investigation of CSCs coincided with novel research into cardiomyogenesis irrespective of source, demonstrating new cardiomyocyte formation in adult human myocardium, although at a low rate of ~1% per annum, reduced to ~0.5% with age (31), compared with ~15% per annum, and ~4% per annum in endothelial and mesenchymal cells, respectively, in the human heart (32). This low rate of cardiomyogenesis is consistent with the essentially absent contractile myocardial repair seen in patients post-MI and with progressive ischaemic heart failure, but directly contrasts with claims of high cardiomyogenesis rates in some CSC publications, some of which were later withdrawn (33). Thus, the current overall consensus is that while CSCs may have cardiomyogenic potential in certain contexts, it is very limited and not exploitable clinically, particularly post-MI (34).

While these complex, often-controversial events unfolded over CSC cardiomyogenic potential, relatively little attention was paid to CSC generation of both endothelial cells and vascular smooth muscle cells. These capabilities have been consistently recognised, from their first identification and repeatedly thereafter by multiple independent research groups (11, 13–15, 20, 29, 30, 35–37). This lesser interest was natural given the well-established ability of vascular cell populations to expand *in situ*, in stark contrast to cardiomyocytes. However, CSC vascular regenerative potential has been shown consistently, including in studies arguing against CSC cardiomyogenesis (20, 29, 37), so is a research avenue separate from the aforementioned controversy.

With the study of CSCs in cardiomyocyte regeneration shifting focus to secretome-based mechanisms, it is worth reviewing the evidence and considering how such paracrine actions may aid cardiac cells beyond cardiomyocytes, in view of evidence about the proportions of cardiac cells that are endothelial (38) and cardiac fibroblast roles in cardiac tissue maintenance (39). The possibility that CSC cell transplant benefits were mediated by paracrine actions was raised quite early in the progress of CSC study (40), with simultaneous articles soon after presenting data and reviewing the understanding to date of paracrine actions of mesenchymal stem cells (MSCs) (41) and CSCs (42), the latter identifying IGF-1 and HGF signalling in CSCs. Increased expression of IGF-1 by CSCs was then shown to increase cardiomyocyte survival *in vitro* (21) and *in vivo* (23, 43).

Increased Notch pathway signalling in a GM mouse model increased CPC expression of TBG-β1 and VEGF, with associated increased myocardial capillary density (44), consistent with the pro-angiogenic actions of those growth factors. Examination of Sca1-positive CSCs found the expression of EGF, TGF-β1, IGF-1, IGF-2, MCP-1, HGF, and IL-6 (45), illustrating that comparable growth factors are expressed in CSCs isolated using different surface markers [the aforementioned studies used c-kit (42, 44)]. This is emphasised by HGF, IGF, and VEGF expression in another class of CSCs, the cardiosphere-derived cells (named for their method of isolation), a type trialled clinically (46).

In summary, while CSC cardiomyogenesis is currently not considered a feasible route for translation, the supportive functions of CSCs—aiding cardiomyocyte survival and microvascular expansion—remain of interest, particularly in diffuse non-infarction cardiac pathologies. Against this background, we consider potential CSC roles in myocardial effects of cancer therapy-related cardiotoxicity.

## Effects on Cardiac Stem Cells of Cardiotoxic Therapies

**Figure 1:**
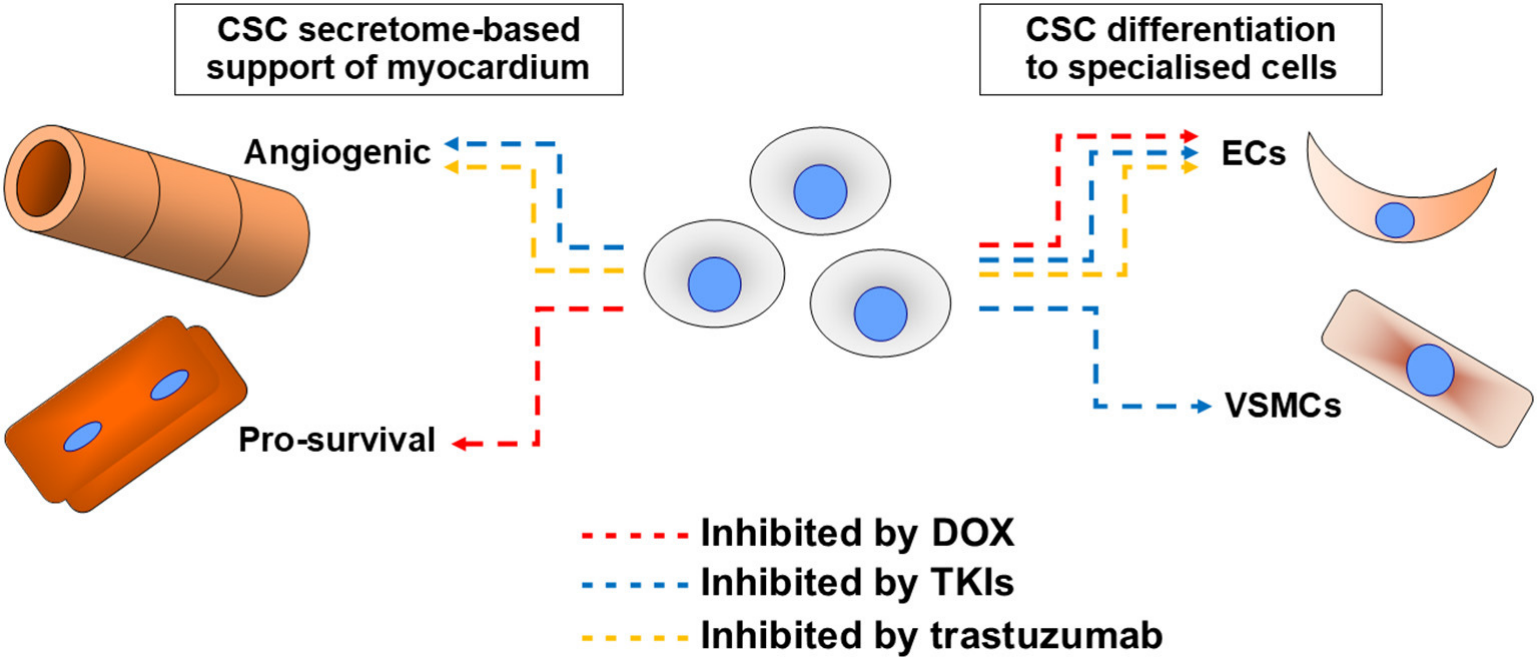
Summary of cardiotoxin effects on cardiac stem cell (CSC) abilities. Impacts of the most examined cardiotoxin doxorubicin (DOX, red), tyrosine kinase inhibitors (TKIs, blue), and trastuzumab (orange) on CSC abilities to support myocardium *via* secretome support of angiogenesis or cell survival (most critically cardiomyocyte survival), or *via* their differentiation into endothelial cells (ECs) or vascular smooth muscle cells (VSMCs).

With the background of CSC translational potential changing dramatically, the more the niche field of examining CSC roles in the pathogenesis of cardiotoxicity underwent related changes in focus. Initial work presupposed any cardiotoxin impacts to directly impact on cardiomyogenesis potential, whereas later work analysed impacts in view of a broader range of roles. Cardiotoxins examined to date are anthracyclines, trastuzumab, and TKIs, with no studies found examining others in CSCs (Figure 1).

The first two studies were published near-simultaneously in 2009–2010 (35, 47), both focusing on DOX effects. The first identified that DOX reduced CSC viability and proliferation both *in vitro* and *in vivo*, but also identified increased markers of CSC differentiation (to both cardiomyocyte and vascular cell lineages) *in vitro* (47). Injection of exogenous CSCs directly into the myocardium reduced DOX-induced myocardial injury, in terms of myocardial fibrosis and wall thickness loss, with associated improved cardiac functional parameters, although the mechanisms were undetermined, so the role(s) of *in situ* CSCs in cardiotoxicity pathogenesis remained uncertain (47). The second study used a model of DOX application in early life, then assessed long-term effects on myocardial homeostasis, to identify damage seen in the long term, perhaps the most likely way that CSCs are involved in cardiotoxicity pathogenesis (35). Notable effects were reduced capillary density and VEGF levels, increased vulnerability to MI in adulthood (infarct sizes in standard MI models were larger post-DOX), and reduced CPC numbers, either infiltrating infarct borders or in uninjured post-DOX hearts (35). A finding contrasting with the prior study (47) was that DOX reduced differentiation, particularly to endothelial cells (35). These findings suggest a role for CSCs in microvasculature formation, a possible way for CSC damage to manifest as long-term cardiotoxicity.

It is notable that DOX cardiotoxicity can develop over a range of time periods, acute (within 2 weeks of treatment completion), early-onset chronic (within 1 year), and late-onset chronic (years or decades later) (48). Loss of CSCs is unlikely to contribute to acute effects, excepting loss of CSC paracrine support for injured cells, but their contribution to angiogenesis could be linked to early- or late-onset chronic effects, respectively. The different rates of cardiotoxicity development in paediatric and adult patients are also notable (49), with cardiomyocyte apoptosis a feasible target for CSC-based protection and CSC protection against this already demonstrated (21).

Another study of DOX in CSCs (albeit those with a phenotype overlapping with MSCs) identified DOX upregulated the stromal cell-derived factor-1/CXC chemokine receptor-4 (SDF1/CXCR4) signal pathway, protecting cells against DOX damage and increasing their migration (50). The SDF1/CXCR4 pathway is upregulated in MI (51) or diffuse myocardial injury (15) and is known to upregulate migration of stem/progenitor cells to injured myocardium (52). Furthermore, blockade of this signalling in CSCs severely reduces their integration into the injured myocardium (15). The pathway also plays a key role in angiogenesis development, *via* a range of different stem/progenitor cells, including endothelial progenitors and MSCs [for review, see (53)].

These combined findings show acute CSC responses to DOX-induced cardiotoxicity, with migration to injured tissue and differentiation (thus, contributing to angiogenesis in damaged tissue), but raises the question on why longer-term depletion of CPCs was seen by Huang et al. (35). There are two possible explanations for this: the first is ongoing mobilisation of the stem cell pool leads to eventual depletion, a phenomenon seen in stem cells that can be induced to generate a prematurely aged phenotype (54). The other explanation is attritional CSC loss from direct DOX toxicity: depleted CSC populations can maintain an acute response, but suffer a critical impairment of their ability to support myocardial homeostasis in the long-term. While SDF application attenuated DOX-induced acute cardiotoxicity (50), whether this impacted on CSC ability to protect against long-term cardiotoxic effects was not studied.

Examination of the mechanisms of acute DOX toxicity in CPCs identified calcium-linked autophagy signalling as playing an important role (55). This is a DOX toxicity mechanism common to CPCs and cardiomyocytes (55, 56), with the underlying generation of mitochondrial oxidative stress also common to DOX toxicity in both cells (49, 56). Rapamycin application reduced cytosolic calcium accumulation in CPCs, along with indications of reduced mTOR signalling (55). Looking at the complexities of developing cardioprotective treatments that also avoid any interference with the—essential—anti-cancer activity of DOX, this overlap in cell death mechanisms is fortuitous, as treatment (perhaps *via* mTOR manipulation) that provides acute protective benefits to cardiomyocytes could also provide long-term benefits *via* CSC protection.

Further evidence of the value of targeting oxidative stress to prevent DOX toxicity in CSCs was shown by the protection of CSCs *in vitro* by encapsulation in a superoxide dismutase-loaded alginate, which averted DOX-induced metabolic alterations and apoptosis (57). A study into the cardioprotective effects of bergamot citrus extract (with known antioxidant effects) against DOX-induced cardiotoxicity in adult rats examined CSCs isolated after *in vivo* treatment: DOX caused significant intranuclear accumulation of reactive oxygen species and reduced CSC numbers *in situ* (58). Bergamot antioxidant treatment significantly attenuated these effects on CSCs, while also reducing DOX-induced cardiomyocyte apoptosis and prevented the cardiac functional damage of DOX (58). Collectively, these findings support the value of antioxidant treatment of acute DOX-induced cardiotoxicity as a protection for CSCs that will also benefit cardiomyocytes.

Although the initial focus for investigation of cardiotoxins in CSCs was DOX, some recent work examined cardiotoxic impacts of other anticancer therapies, particularly TKIs. Treatment of adult rats with imatinib mesylate, unlike DOX, did not cause fibrosis, or loss of cardiomyocyte tissue volume, although cardiomyocyte apoptosis was increased and densities of myocardial capillaries and arterioles were reduced (59). Imatinib reduced CPC numbers *in situ*, and was shown *ex vivo* to reduce both CPC viability and proliferation, along with repressing CPC ability to differentiate (59). The ability of CPCs to protect cardiomyocytes from apoptosis (21) could give valuable protection to cardiomyocytes from imatinib-induced injury (59). In the longer term, with TKI cardiotoxicity significantly damaging cardiac microvasculature, the CPC contribution to repair and regeneration of these vessel networks is of great value, and its loss would worsen disease prognosis.

Only a very little published research has focused on cardiotoxin impacts on another myocardial cell type, cardiac microvascular pericytes, which also have valuable pro-regenerative pro-angiogenic and paracrine actions (60). One such study examined the effects of the TKI sunitinib, finding that the drug caused cardiac microvascular dysfunction and reduced blood flow, with a loss of pericytes that increased with sunitinib treatment progress (10). Another study reaffirmed the toxicity of sunitinib to pericytes, linking it to the mitochondrial deacetylase sirtuin-3 (61). The more recent introduction (since 2000) of TKIs means that the long-term sequelae of their use are not yet fully apparent (particularly in childhood cancers, for which their use was commenced more recently), although comprehensive assessment should consider the myocardial roles of both pericytes and CSCs, along with the impacts of cardiotoxins on both cell types.

We can turn the tables by moving from cardiotoxins damaging CSCs to considering stem cell paracrine abilities as potential anti-cardiotoxin treatments. One study identified protection by stem cell secretome (human amniotic fluid stem cell secretome) of mouse cardiomyocytes *in vitro* against DOX-induced toxicity (62). Further study of this protection found that one effect increased CPC proliferation, with associated increased angiogenesis: no CPC differentiation was seen, although the authors suggested their data supported a local paracrine role for CPCs, but this was not definitively shown (63). This group advanced their work by examining the protection given by human CPC secretome (specifically secreted exosomes) against DOX and trastuzumab: intravenous injection of exosomes reduced damage from DOX and trastuzumab therapy to cardiac function (fibrosis and impaired cardiac functional parameters), also lowering reactive oxygen species in isolated cardiomyocytes treated with DOX and trastuzumab (64). Although trastuzumab did not impact on human cardiosphere-derived cell survival or proliferation, it impaired their ability to form microvascular networks or to commence cardiomyogenic differentiation *in vitro* (65). Furthermore, when cells were applied *in vivo* post-MI, trastuzumab co-application reduced their angiogenesis and associated cardiac functional improvement (65).

CSC secretome-based treatments, potentiating angiogenesis and attenuating cardiomyocyte apoptosis, would complement current anti-cardiotoxicity treatments, focused around the use of ACE inhibitors and beta-blockers to control workload in a weakened heart (66). No examination of ACE-inhibitor or beta-blocker effects on CSCs could be identified, the closest being beta-blockade improving the regenerative action of MSCs post-MI (67).

## Future Directions and Conclusion

Understanding of cardiotoxicity continues to advance, with human cardiomyocytes derived from induced pluripotent stem cells showing great promise [for review, see Ref. (68)]. A striking finding was of cardiac DOX sensitivity at the individual-patient level being reproduced in these cells (69). These developments are very encouraging and rightly reflect a focus on the primacy of cardiomyocytes in cardiotoxicity development. It is, however, important that this progress is complemented by studies considering the other cell types that comprise myocardial tissue and contribute to its maintenance, particularly as cardiomyocyte loss or recovery is partly dependent on these cells.

With ongoing work examining impacts of both older and more novel anticancer therapies on myocardial microvascular cells and tissue (9, 70), indicating that these impacts play key roles in cardiotoxicity development, along with research examining TKI effects on cardiac fibroblasts (71), this essential broader understanding is being built, but much more remains be done.

An important point to stress, the need for this emphasis hopefully illustrated by the brief recap of the CSC-cardiomyogenesis controversy, is what the role played by CSCs in myocardial homeostasis post-cardiotoxicity would likely be. While cardiotoxins destroying or damaging CSCs would indeed diminish the previously assumed function of CSCs as a source of cardiomyogenesis, their support of damaged cardiomyocytes or contribution to new microvasculature formation would be similarly diminished. Therefore, with their recognised potential in these latter areas, determining cardiotoxin impacts on CSCs is a valuable aspect of understanding the long-term pathophysiology of post-cancer treatment cardiotoxicity.

Future developments with the most promise are those harnessing the CSC potential as generators of paracrine protective therapy, either as exogenously generated products applied to injured hearts or as *in situ* mediators. With the *in situ* CSC population sparse (11, 36) and its function declining with age (72), the former route shows more potential as a vector to aid cell survival, microvascular repair or network expansion, with all of these offering clear potential benefits in the drive towards ameliorating the long-term damage wrought by cardiotoxins.

## Author Contributions

The manuscript was written and edited by AS.

## Conflict of Interest

The author declares that the research was conducted in the absence of any commercial or financial relationships that could be construed as a potential conflict of interest.
